# Adipocyte DIO2 Expression Increases in Human Obesity but Is Not Related to Systemic Insulin Sensitivity

**DOI:** 10.1155/2018/2464652

**Published:** 2018-07-15

**Authors:** David Bradley, Joey Liu, Alecia Blaszczak, Valerie Wright, Anahita Jalilvand, Bradley Needleman, Sabrena Noria, David Renton, Willa Hsueh

**Affiliations:** ^1^Diabetes and Metabolism Research Center, Division of Endocrinology, Diabetes & Metabolism, Department of Internal Medicine, Wexner Medical Center, Ohio State University, Columbus, OH, USA; ^2^Center for Minimally Invasive Surgery, Department of General Surgery, Wexner Medical Center, Ohio State University, Columbus, OH 43210, USA

## Abstract

Deiodinase type II (D2), encoded by *DIO2*, catalyzes the conversion of T4 to bioactive T3. T3 not only stimulates adaptive thermogenesis but also affects adipose tissue (AT) lipid accumulation, mitochondrial function, inflammation, and potentially systemic metabolism. Although better defined in brown AT, the precise role of *DIO2* expression in white AT remains largely unknown, with data derived only from whole fat. Therefore, the purpose of this study was to determine whether subcutaneous (SAT) and visceral (VAT) adipocyte-specific gene expression of *DIO2* differs between obese and lean patients and whether these differences relate to alterations in mitochondrial function, fatty acid flux, inflammatory cytokines/adipokines, and ultimately insulin sensitivity. Accordingly, adipocytes of 73 obese and 21 lean subjects were isolated and subjected to gene expression analyses. Our results demonstrate that obese compared to lean human individuals have increased adipocyte-specific *DIO2* expression in both SAT and VAT. Although higher *DIO2* was strongly related to reduced fatty acid synthesis/oxidation and mitochondrial function, we found no relationship to proinflammatory cytokines or insulin resistance and no difference based on diabetic status. Our results suggest that adipocyte-derived *DIO2* may play a role in weight maintenance but is likely not a major contributor to obesity-related insulin resistance.

## 1. Introduction

In spite of growing recognition, the obesity epidemic continues unabated in the United States (US). Over 2/3 of the US adult population is now considered overweight or obese [1, 2]. Obesity contributes to excess morbidity and mortality and adversely affects nearly every organ in the body [3]. With a growing appreciation for the negative health consequences, current treatment options for obesity and its complications remain insufficient and the underlying mechanisms behind obesity-induced complications have yet to be elucidated. Obesity fundamentally results when caloric intake exceeds total energy expenditure [4]. The role of nonshivering or adaptive thermogenesis (the generation of heat through uncoupling of mitochondrial respiration) in total energy expenditure is now being appreciated [5], with obese individuals demonstrating a reduced adaptive thermogenic capacity [6].

Deiodinase type II (D2) is encoded by the gene *DIO2* and catalyzes the conversion of 3,5,3′,5′-tetraiodothyronine (T4) to bioactive 3′,3′,5′-triiodothyronine (T3) by outer ring 5′-deiodination. Cold exposure increases circulating levels of 3′,3′,5′-triiodothyronine (T3) and stimulates catecholamine-induced adaptive thermogenesis [6, 7]. Although the precise role of *DIO2* expression and D2 activity in white adipose tissue (WAT) in contributing to the pathogenesis of human obesity and obesity-related comorbidities, such as type 2 diabetes (T2D), remains largely unknown, recent studies indicate a potential impact. Both polyphenols [8] and bile acids (BAs) [9] increase expression/activation of *DIO2*, improve mitochondrial respiration in BAT and skeletal muscle, and enhance weight loss. In addition, polymorphisms in *DIO2* have been linked to increased insulin resistance, T2D risk, and poor glycemic control [10–12]. Thyroid hormone has major effects on lipid metabolism by activating hepatic and AT lipogenesis via induction of enzymes involved in fatty acid synthesis, including acetyl CoA carboxylase (ACC) and fatty acid synthase (FASN) [13], increasing fatty acid *β*-oxidation though carnitine palmitoyl transferase (CPT) [14, 15], and enhancing lipolysis in animal models [16]. Although gene expression of *DIO2* in whole fat was recently found to be lower in AT of obese patients [17], adipocyte-specific expression and its relationship to mitochondrial function and fatty acid oxidation/synthesis in lean and obese human AT are not known.

Obesity is also associated with a state of low-grade chronic inflammation that contributes to varied comorbidities, including T2D [3], and the balance between pro- and anti-inflammatory mediators and immune cells in AT maintains whole-body metabolism [18]. Thyroid hormone is a potent, but controversial, immunomodulatory factor [19–21], with gene targeting strategies in mice demonstrating an obligate role for thyroid hormone in immune cell development [22]. While an underlying proinflammatory state increases D2 activity and can affect lymphocyte proliferation [23], no human study to date has assessed the relationship between adipocyte *DIO2* expression and pro- and anti-inflammatory factors and its possible impact on glucose homeostasis.

Therefore, the main purpose of this study was to test the hypothesis that SAT and VAT adipocyte gene expression of *DIO2* differs between obese and lean human AT and that these differences will relate to (1) insulin resistance and (2) expression of genes involved in mitochondrial function, fatty acid flux, and inflammation.

## 2. Materials and Methods

### 2.1. Selection and Description of Participants

Ninety-four consecutive eligible patients (BMI 18–40 kg/m^2^; age 21–75 y) who were scheduled to undergo elective abdominal surgery at the Center for Minimally Invasive Surgery or University Hospital East at the Ohio State University (OSU) Wexner Medical Center in Columbus, OH, participated in this study. The study was approved by the OSU Institutional Review Board (IRB). All participants provided written informed consent and completed a comprehensive medical evaluation prior to enrollment. Potential participants who were current smokers, had evidence of end-stage renal or liver disease, had a history of prior organ transplantation, were on chronic pharmacologic steroid or anti-inflammatory use, and had a history of neoplastic disease or chemotherapy within the prior year, acquired immune deficiency syndrome, or >10% body weight loss within 3 months of enrollment were excluded.

### 2.2. Study Design and Experimental Procedures

VAT biopsies were obtained from obese (*n* = 73, age 44.8 ± 11.9 y, BMI 46.7 ± 11.0 kg/m^2^) and lean (*n* = 21, age 47.8 ± 11.6 y, BMI 23.3 ± 1.5 kg/m^2^) patients during elective surgery, either Roux-en-Y gastric bypass or sleeve gastrectomy in obese subjects and elective cholecystectomy, hernia repair, Nissen fundoplication, or Heller procedure for achalasia in lean subjects. SAT biopsies were obtained from all the lean patients and a subset of the obese patients (*n* = 35, age 43.3 ± 11.2 y, BMI 48.0 ± 8.5 kg/m^2^) (Table 1). AT (~10 grams) was obtained at surgery, rapidly transferred on ice, and then processed and fractionated within 15 minutes of obtaining the sample. Briefly, adipose for cell fractionation was minced, collagenase-digested, and fractionated into adipocytes and stromal vascular fractions (SVF) as previously described [24]. Adipocytes were immunodepleted using a CD45 antibody and then flash-frozen, and gene expression was determined using real-time quantitative polymerase chain reaction (qRT-PCR).

#### 2.2.1. qRT-PCR

For qRT-PCR, adipose and adipocytes were analyzed for specific immune cell lineages (Cd3, Emr1) to assess T-cell and macrophage infiltration, respectively. Adipocyte RNA was analyzed for relative expression of *DIO2*, a gene associated with endocrine function (*LEPTIN*, *ADIPOQ*), mitochondrial function (cell death activator (*CIDEA*), ATP synthase (*ATP5A*), and carnitine palmitoyltransferase 1B (*CPT1B*)), fatty acid *β*-oxidation (acetyl CoA dehydrogenase (*ACADM*)), fatty acid synthesis (acetyl-CoA carboxylase (*ACC2*), fatty acid synthase (*FASN*), and diglyceride acyltransferase (*DGAT*)), adipocyte function (PPARgamma (*PPARγ*)), innate immunity (*NLRP3*), and select proinflammatory or anti-inflammatory cytokines or chemoattractants (tumor necrosis factor alpha (*TNFα*), interleukin-1*β* (*IL-1β*), and plasminogen activator inhibitor-1 (*PAI-1*)). Adipocyte RNA was reverse-transcribed and amplified with TaqMan primer/probes (Life Technologies) or SYBR Green Primers (Sigma). RNA expression was normalized to *PPIA*. Gene expression values are shown as the fold change, defined by 2-ddCT.

#### 2.2.2. Analyses of Blood Samples

Plasma glucose, insulin, adiponectin, and leptin concentrations were measured by using enzyme-linked immunosorbent assays (Millipore, Billerica, MA).

#### 2.2.3. Calculations

*(1) Insulin Sensitivity and β-Cell Function*. The homeostasis model assessment of insulin resistance (HOMA-IR) was used to determine the degree of insulin resistance in the subjects. The HOMA-IR accounts for fasting insulin levels relative to prevailing glucose levels and correlates well with more sensitive measures of insulin resistance such as the hyperinsulinemic-euglycemic clamp [25, 26]. *β*-Cell function was determined by the HOMA-*β*, as previously described [26].

### 2.3. Statistical Analysis

Data were examined for normality according to the Shapiro-Wilk criteria and homogeneity of the variance by Levene's test. Groups were compared by one-way ANOVA for normally distributed variables and Mann–Whitney *U* test for nonnormally distributed data. Pearson's correlation for variables with normal distribution and Spearman's correlation for variables with nonnormal distribution were calculated to assess association between variables. Multivariate linear regression analysis with independent variables of age, gender, and BMI was further calculated in a stepwise fashion. All data are presented as means ± standard deviation unless otherwise noted.

## 3. Results

### 3.1. Characteristics of Patients

Demographic data, metabolic variables (fasting serum glucose, insulin, leptin, and adiponectin), and measures of insulin resistance and *β*-cell function are shown in Table 1. Overall, lean patients had lower BMI, fasting glucose, insulin and leptin levels, and HOMA-IR and HOMA-B scores. Lean patients had higher fasting serum adiponectin. There was no difference in age between the lean and obese patient groups.

### 3.2. Adipocyte Gene Expression of DIO2 Is Increased in Obese Human VAT and SAT but Is Not Related to Diabetic Status or Insulin Sensitivity

**Figure 1:**
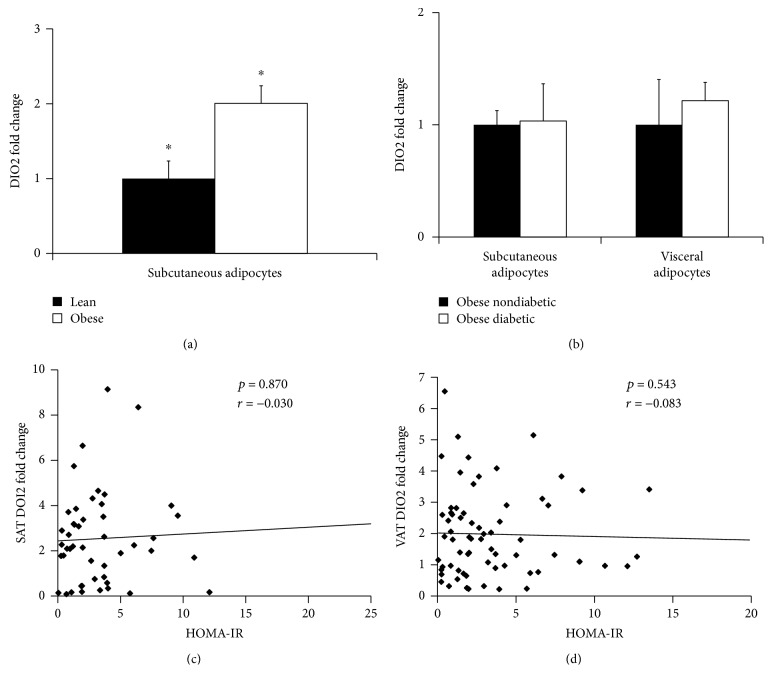
(a) Adipocyte expression of *DIO2* in subcutaneous (SAT) and visceral (VAT) adipose tissue of lean and obese subjects and (b) obese diabetic and obese nondiabetic subjects analyzed by one-way ANOVA. Data presented as mean ± SEM. ^∗^*p* < 0.04. Correlation analyses (Pearson/Spearman) between adipocyte gene expression of *DIO2* in SAT and VAT with HOMA-IR (c, d). HOMA-IR values used for correlation analyses were available for a subset of patients (*n* = 16 lean and *n* = 56 obese).

Although gene expression of whole fat *DIO2* was recently found to be lower in VAT and SAT of obese patients [17] which contain different mixtures of adipocytes and a variety of immune cells, the expression of *DIO2* specifically by the adipocyte is unknown. We thus determined the gene expression of *DIO2* in human obese and lean SAT and VAT adipocytes by qRT-PCR. We observed a significant increase in adipocyte *DIO2* gene expression in obese compared to lean SAT (1.56 ± 0.25 versus 3.03 ± 0.35; *p* = 0.002) and VAT (1.36 ± 0.28 versus 2.12 ± 0.16; *p* < 0.04) (Figure 1(a)). In addition, SAT *DIO2* gene expression was directly associated with BMI (Figure 1(b)). As a significantly greater proportion of lean patients were male (71%) and a greater proportion of obese patients were female (82% in those undergoing VAT biopsies and 77% in those undergoing SAT biopsies), we compared VAT and SAT adipocyte *DIO2* expression by gender. There was no difference between women and men in either visceral (1.9 ± 0.2 versus 2.0 ± 0.2, *p* = 0.938) or subcutaneous adipocyte DIO2 expression (3.0 ± 0.5 versus 2.3 ± 0.5, *p* = 0.270).

Polymorphisms in *DIO2* have been linked to increased insulin resistance, T2D risk, and poor glycemic control [10–12], and T3 has been noted to have effects on mitochondrial function and energy expenditure, both of which can affect metabolic risk. However, we found no difference in adipocyte *DIO2* gene expression between obese diabetic and nondiabetic patients in either SAT (3.11 ± 1.00 versus 3.01 ± 0.38, *p* = 0.465) or VAT (2.44 ± 0.33 versus 2.01 ± 0.81, *p* = 0.455) (Figure 1(d)). In addition, neither SAT (Figure 1(c)) nor VAT (Figure 1(d)) *DIO2* correlated with HOMA-IR, even after adjusting for BMI, age, and gender with multivariate linear regression (SAT: *p* = 0.701; VAT: *p* = 0.797).

### 3.3. Adipocyte Gene Expression of DIO2 Is Related to Fatty Acid Synthesis/Beta-Oxidation and Mitochondrial Function

We next determined whether adipocyte *DIO2* expression is related to expression of genes involved in fatty acid flux and mitochondrial function (Table 2). Overall adipocyte VAT and SAT *DIO2* gene expression was inversely related to markers of mitochondrial function (*CIDEA*, *ATP5A*, *PGC1α*, and *CPT1B*), fatty acid oxidation (*ACADM*), and fatty acid synthesis (*ACC2*, *FASN*, and *DGAT*). *DIO2* expression was inversely related to the anti-inflammatory genes *ADIPOQ* and *PPARgamma* in both VAT and SAT, but of the proinflammatory genes, only *PAI-1* had a significant relationship to *DIO2* expression. There were no significant correlations with *LEPTIN*, *TNFα*, *NLRP3*, or *IL-1β*.

## 4. Discussion and Conclusions

The main purpose of this study was to test the hypothesis that SAT and VAT adipocyte gene expression of *DIO2* differs between obese and lean human AT and that these differences relate to (1) markers of mitochondrial function and fatty acid flux, (2) systemic insulin resistance, and (3) pro- and anti-inflammatory adipokines/cytokines. Our data demonstrates that obese individuals exhibit increased adipocyte *DIO2* expression compared to lean individuals in both SAT and VAT. There were strong relationships between higher *DIO2* and reduced fatty acid synthesis/oxidation and mitochondrial function and adiponectin and PPAR*γ*, and there was no relationship to inflammatory markers except for PAI-1. The clear lack of an association with insulin resistance and the finding of no difference in gene expression between obese diabetic and nondiabetic subjects suggest that adipocyte-derived *DIO2* is not a major contributor to glucose homeostasis.

An imbalance between caloric intake and energy expenditure results in weight gain and ultimately over time in obesity [4]. Although traditionally energy expenditure has been defined as the summation of energy expended through physical activity, resting or basal metabolic rate, and nutrient breakdown, a more prominent role for nonshivering or adaptive thermogenesis is now being appreciated. Adaptive thermogenesis is a component of energy expenditure whereby mammals generate heat through the uncoupling of mitochondrial respiration in brown adipose tissue (BAT) [5]. An expansive body of literature now highlights the role of adaptive thermogenesis and reduced thermogenic capacity in human obesity [6]. However, while the contribution of BAT to adaptive thermogenesis is well studied, the “beiging” of adipocytes within white adipose tissue (WAT) also appears to be an important component to adaptive thermogenesis. Beige adipocytes are induced by cold and/or catecholamine-induced adrenergic stimulation and lead to augmented lipolysis of AT, increased adaptive thermogenesis, and higher total energy expenditure [27]. Cold exposure also increases the levels of biologically active T3, which enhances mitochondrial function and amplifies catecholamine-induced adaptive thermogenesis [6, 7].

Type II iodothyronine deiodinase (D2) is encoded by the gene *DIO2* whose major function is to enzymatically convert thyronine (T4) to T3 [28]. In healthy human subjects, ~70% of the extrathyroidal production of T3 is mediated by D2 [29]. Although D2 is expressed in a large number of cell types including chondrocytes and osteoblasts [30], cochlear and retinal cells [31], and tanycytes in the walls of the third ventricle of the brain [32], among many others [9], the potential role of AT-derived D2 in metabolic disease is unclear. Dietary supplementation with the bile acid cholic acid in mice reverses HFD-induced weight gain by activating D2 and converting T4 to T3 through TGR5 receptor activation. This activation results in increased expression of factors involved in mitochondrial biogenesis, oxidative phosphorylation (PGC-1*α*, CPT-1, UCP-1, and UCP-3), and fatty acid lipolysis [33], which subsequently improve systemic metabolism in murine models. In fact, the metabolic effects of BAs are attenuated in DIO2^−/−^ mice [9]. Although gene expression of *DIO2* in whole fat was reported to be lower in VAT and SAT of obese patients [17], the expression by adipocytes and the relationship between *DIO2*, mitochondrial function, and fatty acid oxidation/synthesis in lean and obese AT remain unknown.

In contrast to that observed in whole fat [17], we now report that *DIO2* expression in isolated adipocytes is actually higher in human obesity and that increased *DIO2* is related to lower mitochondrial gene expression and lipid oxidation, potentially consistent with its strong correlation with BMI in SAT. However, we found no relationship with systemic insulin resistance, suggesting that *DIO2* from the adipocyte likely has minimal effects on human insulin sensitivity and glucose metabolism. In mature adipocytes, mitochondrial dysfunction reduces fatty acid oxidation [34] and promotes lipid accumulation [35]. In a state of chronic nutrient excess, as seen in obesity, these sequelae occur through the overproduction of reactive oxygen species (ROS). Both HFD feeding and hyperglycemia increase ROS production in mouse adipocytes [36, 37], and oxidative stress is amplified in obese human subjects [38]. In turn, excess fatty acids further reduce mitochondrial biogenesis and gene expression, increase ROS, and lead to insulin resistance [34]. In our obese subjects, the strong inverse relationship between adipocyte DIO2 and key markers of mitochondrial function and fatty acid oxidation, coupled with the known effects of thyroid hormone to increase fatty acid *β*-oxidation [14, 15], could indicate a compensatory, but inadequate, response by the adipocyte to restore mitochondrial function and limit harmful lipid accumulation. Future studies, however, are warranted to evaluate these potentially important relationships.

A state of low-grade chronic inflammation is also present in obesity and leads to numerous comorbidities, including T2D [3]. In fact, TNF*α*-mediated ROS accumulation leads to insulin resistance in preadipocytes [39]. Chronic inflammation has also been shown to increase D2 activity. In the current study, we noted negative relationships between *DIO2* and anti-inflammatory gene expression of *PPARγ* and *ADIPOQ* and no association with well-known proinflammatory mediators of insulin sensitivity, including *IL-1β*, *TNFα*, *leptin*, and *NLRP3*. These findings may explain the lack of an association between *DIO2* and insulin resistance and suggest that *DIO2* is not a major driver behind adipocyte-mediated proinflammatory activity and reduced insulin sensitivity.

Our study has several important limitations. Many of our findings are purely associative and may not implicitly constitute a cause-and-effect relationship. In addition, the lack of an association with *DIO2* and insulin sensitivity could be due to insufficient power or subject sample size. However, the clear lack of a correlation in a substantial number of lean and obese human subjects in both SAT and VAT makes a meaningful relationship unlikely. We also measured *DIO2* expression exclusively in AT adipocytes and not specifically in beiging cells, a significant factor in thermogenesis [40] which impacts weight maintenance. Despite no significant difference in age, our lean participants were predominantly male, while the obese subjects were largely female. A comparison of DIO2 expression in both VAT and SAT was similar in male compared to female subjects. In addition, after multivariate adjustment for gender, our findings still consistently failed to demonstrate an important relationship between *DIO2* and IR or *DIO2* and inflammation.

In conclusion, our findings indicate that *DIO2* expression is upregulated in obese subcutaneous and visceral human adipocytes. However, in spite of significant relationships between *DIO2* and reduced mitochondrial function, fatty acid oxidation, and anti-inflammatory adipokines, there was no association with systemic insulin resistance and no difference in diabetic compared to nondiabetic obese human subjects. Our results thus suggest that human adipocyte DIO2 is strongly related to body weight but does not play a major role in overall glucose homeostasis.

## Figures and Tables

**Table 1:** Demographics and clinical characteristics of lean and obese patients.

	Lean subjects (*n* = 21)	Obese subjects with VAT biopsies (*n* = 73)	Obese subjects with SAT biopsies (*n* = 35)
Number of females/number of males (% female)	6/15 (29)	60/13 (82)	27/8 (77)
Age (years)	47.8 ± 11.6	44.8 ± 11.9	43.3 ± 11.2
BMI (kg/m^2^)	23.3 ± 1.5	46.7 ± 11.0^∗^	48.0 ± 8.5^∗^
Fasting glucose (mg/dL)	85.0 ± 13.8	93.4 ± 22.3^∗^	90.9 ± 20.4^∗^
Fasting insulin (*μ*IU/mL)	5.2 ± 5.3	18.8 ± 16.8^∗^	17.3 ± 11.2^∗^
HOMA-IR score	1.1 ± 1.0	4.7 ± 4.7^∗^	4.1 ± 3.1^∗^
HOMA-*β* score	87.5 ± 167.1	236.0 ± 193.8^∗^	243.3 ± 203.9
Plasma adiponectin (ng/mL)	11,294 ± 8448	6453 ± 3723^∗^	6380.3644^∗^
Plasma leptin (ng/mL)	26.9 ± 11.3	47.8 ± 31.9^∗^	55.4 ± 40.7^∗^

Values are means ± SD. BMI: body mass index; HOMA-IR: homeostasis model assessment of insulin resistance; HOMA-*β*: homeostasis model assessment of beta-cell function. ^∗^Value significantly different from the lean value (*p* < 0.05).

**Table 2:** Correlation coefficients (*r*) between adipocyte *DIO2* gene expression (fold change) in visceral (VAT) and subcutaneous (SAT) adipose tissue and gene expression of markers of mitochondrial function, fatty acid oxidation/synthesis, and pro- and anti-inflammatory genes.

	VAT DIO2 gene expression (*n* = 21 lean; *n* = 73 obese)	SAT DIO2 gene expression (*n* = 21 lean; *n* = 35 obese)
*Mitochondrial function genes*		
CIDEA	*r* = −0.365^∗^	*r* = −0.423^∗^
ATP5A	*r* = −0.612^∗^	*r* = −0.601^∗^
PGC1*α*	*r* = −0.493^∗^	—
CPT1B	*r* = −0.436^∗^	*r* = −0.483^∗^

*Fatty acid beta-oxidation genes*		
ACADM	*r* = −0.586^∗^	*r* = −0.549^∗^

*Fatty acid synthesis genes*		
ACC2	*r* = −0.460^∗^	*r* = −0.510^∗^
FASN	*r* = −0.259	*r* = −0.481^∗^
DGAT	*r* = −0.441^∗^	*r* = −0.411^∗^

*Proinflammatory mediator of gene expression*		
IL-1*β*	*r* = −0.091	*r* = +0.045
Leptin	*r* = −0.115	*r* = −0.260
NLRP3	*r* = −0.126	*r* = +0.026
PAI-1	*r* = +0.602^∗^	*r* = +0.324^∗^
TNF*α*	*r* = −0.095	*r* = +0.149

*Anti-inflammatory Mediator of gene expression*		
PPAR*γ*	*r* = −0.656^∗^	*r* = −0.562^∗^
ADIPOQ	*r* = −0.581^∗^	*r* = −0.485^∗^

ADIPOQ: adiponectin; CIDEA: cell death activator; ATP5A: ATP synthase 5A; CPT1B: carnitine palmitoyltransferase 1B; ACADM: acetyl CoA dehydrogenase; ACC2: acetyl CoA carboxylase; FASN: fatty acid synthase; DGAT: diglyceride acyltransferase; PPAR*γ*: PPARgamma; TNF*α*: tumor necrosis factor alpha; IL: interleukin; PAI-1: plasminogen activator inhibitor-1. Innate immunity (NLRP3). ^∗^*p* < 0.05.

## Data Availability

All data related to published results can be supplied upon request to the corresponding author.
